# Roles of Hydrogen Gas in Plants under Abiotic Stress: Current Knowledge and Perspectives

**DOI:** 10.3390/antiox11101999

**Published:** 2022-10-09

**Authors:** Changxia Li, Wenjin Yu, Yuancai Wu, Yongqiang Li

**Affiliations:** College of Agriculture, Guangxi University, Nanning 530004, China

**Keywords:** hydrogen gas, environmental stresses, regulatory pathways, metabolisms, genes

## Abstract

Hydrogen gas (H_2_) is a unique molecular messenger, which is known to be involved in diverse physiological processes in plants, from seed germination to seedling growth to regulation of environmental stresses. In this review, we focus on the role of H_2_ in plant responses to abiotic stresses, such as temperature, osmotic stress, light, paraquat (PQ)-induced oxidative stresses, and metal stresses. In general, H_2_ can alleviate environmental stresses by improving the antioxidant defense system, photosynthetic capacity, re-establishing ion homeostasis and glutathione homeostasis, maintaining nutrient element homeostasis, mediating glucose metabolism and flavonoid pathways, regulating heme oxygenase-1 (HO-1) signaling, and interaction between H_2_ and nitric oxide (NO), carbonic oxide (CO), or plant hormones. In addition, some genes modulated by H_2_ under abiotic stresses are also discussed. Detailed evidence of molecular mechanisms for H_2_-mediated particular pathways under abiotic stress, however, is scarce. Further studies regarding the regulatory roles of H_2_ in modulating abiotic stresses research should focus on the molecular details of the particular pathways that are activated in plants. More research work will improve knowledge concerning possible applications of hydrogen-rich water (HRW) to respond to abiotic stresses with the aim of enhancing crop quality and economic value.

## 1. Introduction

**Figure 1:**
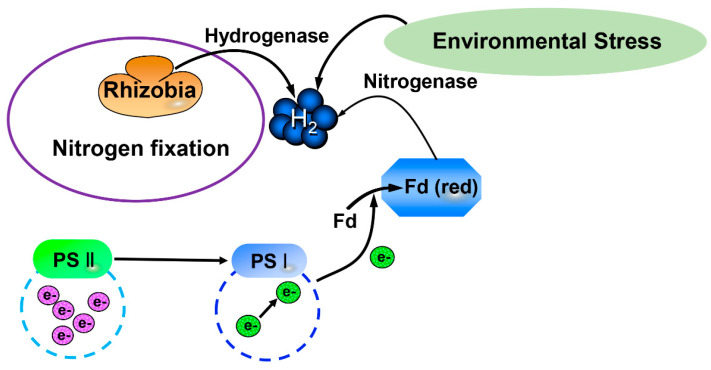
H_2_ production pathway in organisms. H_2_, Hydrogen gas; Fd, Ferredoxin; PS II, Photosynthetic system II; PS I, Photosynthetic system I.

Hydrogen is the most abundant element on earth, comprising ~75% of the mass of the earth. Its molecular form, hydrogen gas (H_2_), is a colorless, odorless and tasteless gas. Since H_2_ was first purified by Robert Boyle in 1671, it has been known as a reducing gas [1]. The production of H_2_ was first observed in bacteria [2] and then in green algae [3]. Sanadze [4] first found that the production of endogenous H_2_ existed in higher plants. The release of endogenous H_2_ has been widely found in higher plants since then [5,6]. It has been established that there are two pathways to produce H_2_ in plant cells, via hydrogenases and nitrogenases [7,8,9]. Briefly, electrons are formed and transmitted in photosynthetic systems. Then, the electrons are activated, which reduces ferredoxin (Fd). Fd (red) can be reoxidized by hydrogenases to form H_2_. Moreover, rhizobia can release H_2_ during the nitrogen fixation process (Figure 1; [7,8,9]). Additionally, environmental stress also promotes the production of endogenous H_2_ in plants Figure 1; [10,11,12].

H_2_ was reported as a therapeutic antioxidant and has become a hotspot in medical research since 2007. Researchers published more and more related papers in areas such as diabetes, organ ischemia-reperfusion injury, atherosclerosis, cancer, hypertension, and other major human diseases [13,14,15,16]. A review by Qian et al. [17] noted that H_2_ was involved in treating some important diseases due to its antiapoptotic and anti-inflammatory effects, including nervous system disorders, liver diseases and metabolic diseases. In plants, H_2_ acts as an important bio-regulator that modulates various physiological processes, including seed germination, seedling growth, adventitious rooting, root elongation, and post-harvesting. For example, Xu et al. [18] demonstrated that H_2_ promoted remarkably seed germination of rice (*Oryza sativa* L.) under salt stress. H_2_ could increase the growth of seedlings of alfalfa (*Medicago sativa* L.), maize (*Zea mays* L.), and Chinese cabbage (*Brassica campestris* spp. *chinensis* L.) [19,20,21]. Adventitious root development in cucumber was improved significantly by H_2_ [22,23]. H_2_ significantly promoted the root length in cucumbers [18]. H_2_ also significantly delayed the ripening process of kiwifruit (*Actinidia chinesis* cv. Huayo) [24].

In nature, plants are constantly challenged by a variety of environmental stresses [25,26]. In plants, numerous growth and development processes, such as root elongation, seedling growth and seed germination, are altered by environmental stresses. For instance, cadmium (Cd) stress significantly inhibited the root elongation and fresh weight of Pak Choi (*Brassica campestris* ssp. *Chinensis*) seedlings [10]. Zhao et al. [27] implied that the growth of maize seedlings was inhibited by aluminum (Al) stress. Oxidative stress usually appears when plants are subjected to various environmental stresses, indicated by an increase of reactive oxygen species (ROS) and responses by antioxidant systems. Chen et al. [28] reported that cucumber (*Cucumis sativus* L.) seedlings suffering from heat stress showed increased malondialdehyde (MDA) and hydrogen peroxide (H_2_O_2_) content and decreased activity of superoxide dismutase (SOD), catalase (CAT), ascorbate peroxidase (APX) and peroxidase (POD) enzymes. Under cold stress, the levels of H_2_O_2_ and MDA in rice seedlings were remarkably elevated [29]. Al stress led to reductions in calcium (Ca), iron (Fe), and magnesium (Mg) uptake in both maize roots and shoots [27]. High light stress effectively blocked the photosynthetic capacity of maize seedlings [21]. Anthocyanin content of radish (*Raphanus sativus* L.) sprouts was decreased significantly by ultraviolet-A (UV-A) irritation [30]. Therefore, it is particularly important to explore the factors that improve the environmental stress-tolerance of plants. An increasing body of evidence suggests that phytohormones contribute to plant response to various environmental stresses. Nakashima et al. [31] summarized that abscisic acid (ABA) and ABA signaling factors may improve tolerance to environmental stresses. Li et al. [32] found that brassinolide enhanced cold stress tolerance of mango (*Mangifera indica* L.) through regulating plasma membrane proteins and lipids. The ethylene signaling pathway was also found to regulate salt stress response [33]. In addition, gas signaling molecules are involved in the regulation of abiotic stress. A review by Siddiqui et al. [34] showed that nitric oxide (NO) plays a vital role in resistance to salt, drought, temperature, ultraviolet-B (UV-B), and heavy metal stress. Hydrogen sulfide (H_2_S) may improve plant tolerance to stresses [35]. Carbon monoxide (CO) was also reported to be involved in the regulation of various environmental stresses [36,37]. H_2_ has emerged as an important antioxidant in plants that can respond to all kinds of environmental stresses such as temperature, light, metals, osmotic, and paraquat (PQ) stresses [6,20,21,27,28,29,30]. For instance, H_2_ could enhance cold tolerance in cucumber seedlings [29]. H_2_ had a positive effect against high light-induced stress in maize seedlings [21]. Toxicity symptoms of Cd-induced in Chinese cabbage were alleviated by H_2_ [20]. H_2_ could enhance tolerance to osmotic stress in alfalfa [11]. The tolerance of PQ-induced oxidative stress was increased by H_2_ in alfalfa seedlings [6]. Moreover, H_2_ can crosstalk with other molecules to respond to various abiotic stresses. Interaction of H_2_ and NO can enhance Al stress via alleviating Al toxicity symptoms in alfalfa [38]. Cross-talk between CO and H_2_ could be involved in drought stress responses [39].

Considering the rapid increase of interest in the role of H_2_ in plants’ response to abiotic stress, this paper summarizes the information about H_2_-mediated abiotic stress and regulatory pathways and mechanisms based on the most recent works in the literature. Additionally, we also briefly consider H_2_-mediated related gene expression.

## 2. H_2_ Confers Plant Tolerance to Environmental Stresses

Plants are invariably exposed to different environmental stresses in the process of growth and development, which has profound effects on crop production [40]. When plants are stressed, a variety of significant physiological changes will appear, such as reduced photosynthesis, ROS production, disturbed ion homeostasis of plant cells, and so on. Previous studies indicated that phytohormones and signaling molecules played an important role in plant resistance to environmental stress [31,33]. Nowadays, it has become increasingly evident that H_2_ enhances plant resistance to environmental stimuli [19,20,21] (Table 1).

### 2.1. Temperature Stress

In general, temperature stress includes cold stress and heat stress [28,29] (Table 1). Purified H_2_ (99.99%, *v*/*v*) was bubbled into 1000 mL Hoagland’s solution (pH 5.87, 25 °C), which was used as 100% hydrogen-rich water (HRW). 50% HRW is diluted from 100% HRW. After the 3-week-old cucumber seedlings were pretreated with 50 and 100% HRW for 7 days, HRW pretreatment remarkably enhanced cucumber seedlings’ heat-tolerance in comparison with heat stress treatment alone when cucumber seedlings were subjected to heat stress for 3 days (42/38 °C), and 50% HRW most effectively mediated heat-tolerance [28] (Table 1). The authors further proved that HRW pretreatment could enhance heat stress tolerance by improving photosynthetic capacity, antioxidant response, and the accumulation of HSP70 and osmolytes in cucumber seedlings [28]. Xu et al. [29] found that with 0.39 mM H_2_ pretreatment, rice seedlings exhibited the maximum prevention of cold-induced growth inhibition and improved cold tolerance (0 °C). They also suggested that endogenous H_2_ might contribute to the enhancement of cold tolerance by increasing photosynthetic capacity, improving antioxidant response, and reestablishing redox homeostasis mediated by miR398 and miR319 expression [29] (Table 1). Along with these studies, we suggest that H_2_ might contribute to the enhancement of temperature tolerance in plants through improving photosynthetic capacity, increasing antioxidant response, promoting the accumulation of HSP70 and osmolytes, and regulating RNA expression that is required for redox homeostasis. As there are few studies about the roles of H_2_ in developing temperature stress tolerance, more work is required on this topic.

### 2.2. Light Stress

The quality and amount of light that plants receive strongly influence their growth and development, but when more light is absorbed than is required for photosynthesis, the excess light can cause stress that can be harmful due to the production of ROS [41]. High light stress may result in stunted growth of maize seedlings, while exogenous H_2_ supplementation could alleviate the phenomenon [21] (Table 1). These results confirmed that H_2_ could alleviate high light stress in maize by decreasing the susceptibility of the PSII (ΦPSII) to photoinhibition and increasing antioxidant response [21]. As one of the main components of light, UV radiation has been recognized as causing light stress on plant growth and development. In general, UV radiation may increase DNA damage, stimulate antioxidant response, induce flavonoid production, and alter the expression of defense-related genes. Su et al. [30] reported that H_2_ significantly blocked the UV-A-induced antioxidant response of radish. Furthermore, H_2_ markedly enhanced UV-A-induced increase of anthocyanin and total phenols contents in radish (high level of anthocyanin), implying that H_2_ could alleviate UV-A radiation by reestablishing reactive oxygen species homeostasis and anthocyanin accumulation [30] (Table 1). The exposure of alfalfa seedlings to UV-B irradiation significantly increased endogenous H_2_ content, suggesting that H_2_ may play an important role in the response to UV-B irradiation [42] (Table 1). The authors suggest that H_2_ conferred tolerance to UV-B irradiation via the manipulation of antioxidant defense and (iso)flavonoids metabolism in alfalfa [42]. Combined with the above results, we suggest that H_2_ will have practical applications for enhancing the temperature tolerance of plants by improving photosynthesis, antioxidant response, and anthocyanin accumulation. These results will open a new window for increasing plant tolerance to light stress. However, whether there are other pathways that help alleviate light stress in response to H_2_ still needs further investigation.

**Table 1:** Overview of the abiotic stresses alleviated by H_2_ and regulatory pathways involved in the process in plants.

Stresses	Plants	Regulatory Pathways	References
Cold	Rice	ROS and antioxidant defense system Photosynthetic capacity	[29]
High light	Maize		[21]
UV-A	Radish	ROS and antioxidant defense system Flavonoid pathway	[30]
UV-B	Alfalfa	Flavonoid pathway	[42]
Hg	Alfalfa	ROS and antioxidant defense system Re-establishing ion homeostasis Re-establishing redox homeostasis	[43]
Cd/Salt	*Hypsizygus marmoreus*	ROS and antioxidant defense system Glucose metabolism	[44]
Cd Cd	Chinese cabbage	ROS and antioxidant defense system	[20]
	Alfalfa	ROS and antioxidant defense system Re-establishing redox homeostasis	[45]
Al	Maize	ROS and antioxidant defense system Photosynthetic capacity Maintaining nutrient element homeostasis	[27]
	Alfalfa	NO production	[38]
Cd	Alfalfa	ROS and antioxidant defense system Re-establishing ion homeostasis Maintaining nutrient element homeostasis	[19]
	Pak Choi	Re-establishing ion homeostasis	[10]
Osmotic	Alfalfa	NO Signaling	[46]
	Alfalfa	ROS and antioxidant defense system HO-1 signaling	[11]
Drought	Alfalfa	ABA	[47]
	Cucumber	CO signaling	[39]
Salt	Arabidopsis	ROS and antioxidant defense system Re-establishing ion homeostasis	[5]
	Rice	ROS and antioxidant defense system Glucose metabolism Re-establishing ion homeostasis	[18]
Cd/Salt	Alfalfa	ABA, ethylene, or jasmonate acid	[12]
PQ	Alfalfa	ROS and antioxidant defense system HO-1 Signaling	[6]

Notes: ROS, Reactive oxygen species; UV-A, Ultraviolet-A; UV-B, Ultraviolet-B; Hg, Mercury; Cd, Cadmium; NO, Nitric oxide; HO-1, Heme oxygenase-1; ABA, Abscisic acid; CO, Carbon monoxide; PQ, Paraquat.

### 2.3. Metals Stress

Metals such as mercury (Hg), Cd, and Al have become major environmental contaminants that restrict plant growth and development. Cui et al. [43] indicated that the growth of alfalfa seedlings was markedly inhibited when exposed to mercury chloride (HgCl_2_), but the effect could be blocked by HRW. They also found that H_2_ attenuated Hg toxicity via reducing Hg accumulation, improving the antioxidant defense system, and reestablishing redox homeostasis [43] (Table 1). In *Hypsizygus marmoreus*, 0.8 mM H_2_ could remarkably reduce Cd toxicity, leading to a significant improvement of mycelial growth and biomass [44] (Table 1). These findings also suggested that H_2_ could be an effective approach for Cd detoxification via enhancing antioxidant response and glucose metabolism [44]. Cd toxicity caused root elongation and seedling growth inhibition in Chinese cabbage, but the addition of 50% saturation HRW significantly alleviated the symptoms that were reported by Wu et al. [20]. Their results also suggested that the improvement of Cd tolerance by HRW was closely associated with reduced Cd uptake and increased antioxidant defense capacities [20] (Table 1). Likewise, root elongation and seedling growth of alfalfa were inhibited by Cd stress, but the appearance of Cd toxicity symptoms was significantly alleviated when 10% HRW was added [45] (Table 1). These findings indicated that H_2_ could improve Cd tolerance, which was consistent with reestablished glutathione homeostasis and a lesser quantity of accumulated Cd [45]. Al stress inhibited the growth of maize seedlings [27] (Table 1). However, 75% HRW markedly promoted root elongation of maize, thereby alleviating Al toxicity [27]. Zhao et al. [27] also concluded that H_2_ could enhance Al tolerance by improving photosynthesis, reestablishing redox homeostasis, and maintaining nutrient homeostasis. Similarly, Chen et al. [38] indicated that H_2_ can alleviate Al-induced alfalfa seedling growth inhibition. However, the difference was that H_2_ improved Al tolerance by reducing NO production and decreasing Al uptake [38] (Table 1). Application of H_2_ could alleviate Cd-induced growth inhibition of alfalfa seedlings [19] (Table 1). Wu et al. [10] showed that Pak Choi exposure to Cd resulted in a rapid increase in endogenous H_2_ production, and exogenous application of HRW reduced a Cd stress-induced phenotype. Their results further indicated that H_2_ operated upstream of the IRT1 transporter and regulated root Cd uptake by controlling plasma membrane-based nicotinamide adenine dinucleotide phosphate (NADPH) oxidase encoded by the *RbohD* gene [10] (Table 1). As mentioned above, H_2_ alleviates metal toxicity mainly by reducing metal accumulation, improving the antioxidant defense system and glucose metabolism, reestablishing redox homeostasis, increasing photosynthesis, maintaining nutrient homeostasis, and reducing NO production. There is a real need for continued research in this area in order to reveal the underlying regulatory mechanisms of H_2_ on metal stresses because these stresses impose considerable constraints on plant growth and development and crop production.

### 2.4. Osmotic Stress

Osmotic stress is one of the most significant abiotic stresses including the effects ofdrought and salinity, and affects virtually every aspect of plant physiology and metabolism. Su et al. [46] found that osmotic tolerance of alfalfa seedlings was closely linked with H_2_ (Table 1). Under polyethylene glycol (PEG)-induced osmotic stress, H_2_ alleviated root inhibition by decreasing lipid peroxidation, and H_2_O_2_ and heme oxygenase-1 (HO-1) were involved in H_2_-induced osmotic stress [11] (Table 1). H_2_-mediated enhancement of alfalfa seedlings’ tolerance to drought stress was also suggested by Jin et al. [47] (Table 1). These responses were supported by Chen et al. [39] (Table 1). H_2_ also could alleviate salt-induced growth inhibition in Arabidopsis (*Arabidopsis thalian* L.) [5] (Table 1). These findings further indicated that H_2_ improved the salt tolerance of Arabidopsis by modulating genes/proteins of zinc-finger transcription factor ZAT10/12, antioxidant defense, and maintenance of ion homeostasis [5]. Similarly, salt stress caused endogenous H_2_ production in germinating rice seeds, and H_2_ pretreatment more or less attenuated salt-induced inhibition of seed germination and seedling growth [18] (Table 1). The authors also suggested that H_2_ alleviated salt stress in rice, which was consistent with sugar metabolism, the ratio of potassium (K) to sodium (Na), and antioxidant capability [18]. Under salt stress, H_2_ improved salt tolerance in *Hypsizygus marmoreus* by increasing glucose metabolism and antioxidant response [44] (Table 1). Zeng et al. [12] also found that endogenous H_2_ production in rice was induced under salt and drought stresses. They suggested that H_2_ alleviated salt and drought stress in rice by modulating the output of hormone signaling pathways [12] (Table 1). In conclusion, H_2_ could enhance the tolerance against osmotic stress by maintaining ion homeostasis, increasing antioxidant capacity, and regulating sugar metabolism.

### 2.5. PQ-Induced Oxidative Stress

Like many environmental stresses, PQ (a methyl viologen family of herbicide widely used to mimic oxidative stress) subjects plants to oxidative damage, leading to excessive ROS production [48]. When alfalfa seedlings were exposed to PQ stress, endogenous H_2_ production and lipid peroxidation were increased [6] (Table 1). The activities of antioxidant enzymes, the level of MsHO-1 transcript and HO-1 activity were increased after exposure to H_2_ [6]. These results indicated that H_2_ might alleviate PQ stress though the antioxidant system and HO-1 signaling [6]. However, only this report has been published to support the suggestion that H_2_ could alleviate PQ stress in plants. Whether there are more pathways by which H_2_ might alleviate the PQ stress still needs further investigation.

## 3. The Regulatory Pathways Abiotic Stress Alleviated by H_2_ in Plants

### 3.1. ROS and Antioxidant Defense System

**Figure 2:**
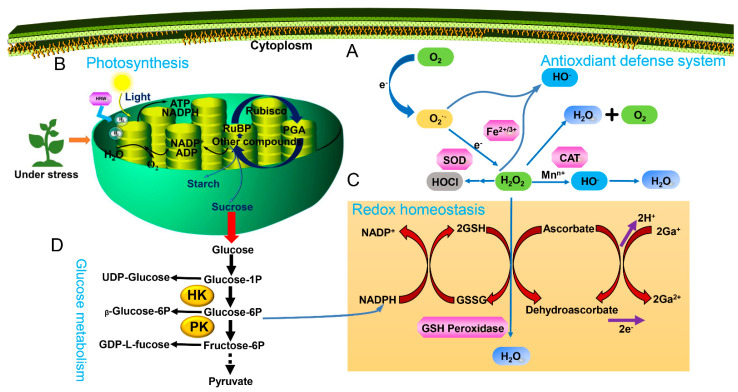
The regulatory mechanism of H_2_-medicated abiotic stress. (**A**), Antioxidant defense system. H_2_ could regulate the antioxidant system by increasing the activities of antioxidant-related enzymes and decreasing ROS accumulation and lipid peroxidation to relieve environmental stresses in plants. (**B**), Photosynthesis, H_2_ could relieve abiotic stresses due to photosynthesis. (**C**), Redox homeostasis. H_2_ could also regulate abiotic stresses via re-establishing redox homeostasis. (**D**), Glucose metabolism. Glucose metabolism is also involved in the regulation of abiotic stresses. H_2_O_2_, Hydrogen peroxide; O_2_^−^, Superoxide anion; O_2_, Oxygen; Water, H_2_O; SOD, Superoxide dismutase; CAT, Catalase; NADPH, Nicotinamide adenine dinucleotide phosphate; ATP, Adenosine triphosphate; RuBP, Ribulose 1,5-bisphosphate; PGA, 3-phosphoglyceric acid; ADP, Adenosine diphosphate; NADP^+^, Nicotinamide-adenine dinucleotide phosphate; Glucose-1P, Glucose-1-Phosphate; Glucose-6P, Glucose-6-Phosphate; Fructose-6P, Glucose-6-Phosphate; HK, Hexokinase; PK, Pyruvate kinase; GSH, Glutathione; GSSG, Oxidized glutathione.

Environmental stress usually triggers ROS production in the chloroplasts, mitochondria, peroxisomes, plasma membranes, endoplasmic reticulum (ER), and the cell wall, and the accumulation of ROS can lead to lipid peroxidation, disruption of the cell membrane, and inhibition of plant growth [48,49]. The ROS mainly comprise oxygen (O_2_), H_2_O_2_, superoxide anion (O_2_^−^), and hydroxyl radical (OH^−−^). H_2_O_2_ and O_2_^−^ are produced in plant cells under abiotic stresses, caused by factors like salt, heat, light, metal stresses and PQ-induced oxidative stress [25,50]. H_2_O_2_ and O_2_^−^ behave like double-edged swords in plant cells; they are beneficial at low concentrations, but damaging at higher concentrations. For example, PQ-induced oxidative stress in alfalfa seedlings resulted in an increase in H_2_O_2_ and O_2_^−^ [6]. UV-A induced significantly increased H_2_O_2_ and O_2_^−^ levels in radish [30]. To avoid ROS-induced cellular injury, plants utilize their antioxidant systems, notably SOD, CAT, CAT and APX, and monodehydroascorbate reductase (MDHAR), to scavenge the ROS [48,49]. SOD, CAT, and APX activities were increased by H_2_ in response to salt stress in rice ([18], Table 1, Figure 2A). Lipids form a major portion of the plasma membrane which envelops the cell and helps it to adapt to the changing environment [51]. Sodium chloride (NaCl) induced ROS overproduction in rice, thereby leading to lipid peroxidation [18]. Salt-induced oxidative stress was alleviated by H_2_ by increasing APX activity and decreasing thiobarbituric acid reactive substances (TBARS) content ([5], Table 1, Figure 1A). H_2_ could enhance the heat-tolerance of cucumber seedlings via increasing SOD, POD, CAT, and APX activities, which reduced the increased MDA and H_2_O_2_ content [28] (Table 1, Figure 2A). The elevated H_2_O_2_ and MDA contents under cold stress were also decreased by H_2_ via significant increases in SOD, POD, and CAT activities [29] (Table 1, Figure 2A). Zhang et al. [28] also found that heat stress resulted in the increase of O_2_^−^ and H_2_O_2_ contents. When H_2_ was added, the enhancement of SOD, CAT, APX and glutathione reductase (GR) activities decreased O_2_^−^ and H_2_O_2_ contents, thereby improving the high light-tolerance of maize seedlings (Table 1, Figure 2A). HgCl_2_ triggered the increase of ROS, which lead to lipid peroxidation in alfalfa, but the adverse effect was reversed by H_2_ by enhancing the activities of SOD, POD and APX enzymes [43] (Table 1, Figure 2A). H_2_ enhanced tolerance of PQ-induced oxidative stress in alfalfa seedlings through increasing the activities of SOD, CAT, POD and APX [6]. Under Al stress, TBARS, O_2_^−^ and H_2_O_2_ levels increased significantly in maize leaves and roots, whereas the increase was suppressed by H_2_ [27] (Table 1, Figure 2A). Simultaneously, H_2_ also played a promoting role in the Al stress-inhibited the activities of POD, SOD, APX and CAT [27]. H_2_ increased significantly the activities of SOD, CAT and GR enzymes, thus decreasing remarkably the levels of O_2_^−^ and H_2_O_2_ in *Hypsizygus marmoreus’* response to Gd and salt stresses [44] (Table 1, Figure 2A). Su et al. [30] reported that H_2_ remarkably blocked UV-A-induced increase of O_2_^−^ and H_2_O_2_ levels though enhancing UV-A-induced decrease of SOD and APX activities (Table 1, Figure 2A). Wu et al. [20] found that H_2_ could improve the tolerance of Cd via enhancing the activities of SOD, POD, CAT and APX and decreasing TBARS content and ROS production in Chinese cabbage (Table 1, Figure 2A). H_2_ prevented ROS accumulation and lipid peroxidation against Cd stress, followed by the increase of SOD, POD, APX and GPX activities [19] (Table 1, Figure 2A). H_2_ could alleviate Cd stress, as indicated by the decrease of TBARS and ROS production [20] (Table 1, Figure 2A). Under PEG-induced osmotic stress, the lipid peroxidation was decreased when H_2_ was administrated [11] (Table 1, Figure 2A).

The plasma membrane which surrounds the entire plant cell plays an important role in interacting with the ever-changing environmental conditions and provides information necessary for the continual survival of the cell [51]. The root activity and plasma membrane integrity were maintained by H_2_ under Al stress [27]. Plasma membrane integrity was maintained by H_2_ via the increase of lipoxygenase (LOC) activity against Cd stress in alfalfa [19]. These results implied that H_2_ could alleviate various environmental stresses via ROS and antioxidant defense systems. However, the molecular mechanisms regulated by H_2_ during this process are still unknown. ROS functions, individual signaling pathways, and critical ROS concentrations regulated by H_2_ against abiotic stress are unclear. Further studies are needed regarding the molecular mechanism of H_2_ in regulating the ROS and antioxidant defense system and ROS functions, individual signaling pathways, and critical ROS concentration regulated by H_2_ under abiotic stresses.

### 3.2. Photosynthetic Capacity

H_2_ could improve the heat-tolerance of cucumber seedlings by mitigating the reduction of heat-induced photosynthetic rate (Pn), stomatal conductance (gs), mesophyll conductance (Gm) and water use efficiency (WUE) and the increase of heat-induced intercellular CO_2_ concentration (Ci) and transpiration rate (E) [28]. Simultaneously, H_2_ also significantly increased PSII maximal photochemistry efficiency (Fv/Fm), effective quantum yield of PSII, electron transport rate (ETR), the excitation capture efficiency of open centers (Fv′/Fm′) and photochemical quenching (qP) during this process [28]. The light absorbed in PSII antennae can be divided into three parts: (1) energy utilized in photosynthesis (P), (2) energy dissipated by photo-protective mechanisms (D) and (3) the excess light energy that is neither utilized nor dissipated (E) [52]. Among them, P reflects the photosynthetic capacity of the entire PSII. Chen et al. [28] also reported that heat induced decreased P and increased D and E, but that H_2_ reversed the effects of heat, suggesting that H_2_ could improve the heat-tolerance of cucumber seedlings by enhancing photosynthetic capacity (Table 1, Figure 2B). Under cold stress, chlorophyll a, b, and total chlorophyll contents of rice seedlings were significantly reduced, which were reversed by H_2_ [29] (Table 1, Figure 2B). Similarly, H_2_ also blocked cold stress-induced reduction of Pn and gs and elevation of intercellular CO_2_ concentration (Ci) [29]. H_2_ could increase significantly and bring rapid recovery of the PSII maximal Fv/Fm and PSII performance index on absorption base (PIABS) response to high light stress which indicated that H_2_ could alleviate effectively high light stress-induced damage to PSII [21]. The authors also found that H_2_ could remarkably reduce the high light-induced damage to the acceptor side, reaction centre, as well as donor side of the photosystem II [21]. H_2_ ameliorated Al stress-inhibited total chlorophyll content, net photosynthesis rate, stomatal conductance and phosphoenolpyruvate carboxylase (PEPC) of maize leaves [27] (Table 1, Figure 2B). Electron transport flux per excited cross section (ETo/CSo) and the number of active PSII reaction centers per excited cross section (RC/CSo) are important here. The decrease of Fv/Fm, PIABS, ETo/CSo, RC/CSo and (trapped energy flux per excited cross section) Tro/Cso caused by Al stress was suppressed when HRW was added to the Al treatment [27]. However, the molecular mechanism of the H_2_-mediated photosynthetic system response to abiotic stress is unclear.

### 3.3. Re-Establishing Redox Homeostasis

Under Hg stress, H_2_ not only differentially strengthened the increased tendencies in the contents of GSH (in particular), GSSG, hGSSGh, and AsA, but also blocked the hGSH and DHA levels [43] (Table 1, Figure 2C). High ratios of GSH/GSSG, hGSH/hGSSGh, and AsA/DHA were exhibited in HRW pretreatment compared with Hg treatment [43]. These data imply that H_2_ was able to alleviate Hg toxicity in alfalfa seedlings via reestablishing glutathione homeostasis [43]. Similarly, H_2_ significantly eliminated the Cd-induced decrease of GSH content and increase of GSSG content [45] (Table 1, Figure 2C). Under Cd stress, a higher ratio of GSH/GSSG responsive for intracellular redox status was exhibited in the HRW treatment [43]. Research showing that H_2_ alleviated abiotic stress via reestablishing redox homeostasis is scarce.

### 3.4. Glucose Metabolism

H_2_ could alleviate salt stress in rice seeds by activating α/β-amylase activity and accelerating the formation of reducing sugar and total soluble sugar [18]. Under Cd and salt stresses, H_2_ enhanced PK activity, suggesting that H_2_-mediated glucose metabolism regulated Cd and salt stresses [44] (Table 1, Figure 2D). Whether H_2_ mediated glucose metabolism during other stresses is unclear.

### 3.5. Re-Establishing Ion Homeostasis

The ratio of potassium (K) to sodium (Na) of shoot and root parts in rice was increased by H_2_ in response to salt stress [18] (Table 1). Under salt stress, H_2_ regulated the antiporters and H^+^ pump responsible for Na^+^ exclusion (in particular) and compartmentation that maintained ion homeostasis in Arabidopsis [5] (Table 1). Hg-induced increase in relative ion leakage was significantly reversed by H_2_ [43] (Table 1). H_2_ reduced Cd uptake of Pak Choi via Ca^2+^ transport across the plasma membrane and apoplastic H_2_O_2_ generation by BcRbohD under Cd stress [10] (Table 1). Metal ion homeostasis was associated with Cd resistance conferred by H_2_ [19] (Table 1). Therefore, in the future, more detailed study is needed of the regulatory mechanisms by which H_2_ alleviated stress through re-establishing ion homeostasis.

### 3.6. Maintaining Nutrient Element Homeostasis

Al stress led to reductions in Ca, Fe, and Mg uptake in both maize roots and shoots, while H_2_ significantly improved the uptake of P and Fe in roots and Ca, P, Fe, and Mg in shoots, implying that H_2_ could alleviate Al stress via maintaining nutrient element homeostasis [27] (Table 1). Under Cd stress, nutrient element homeostasis was closely associated with H_2_-improved Cd tolerance of alfalfa [19] (Table 1). Studies on the abiotic stresses alleviated by H_2_ via maintaining nutrient element homeostasis are scarce, however. Further research is required into the abiotic stresses alleviated by H_2_ and the regulatory mechanisms for maintaining nutrient element homeostasis.

### 3.7. Flavonoid Pathway

**Figure 3:**
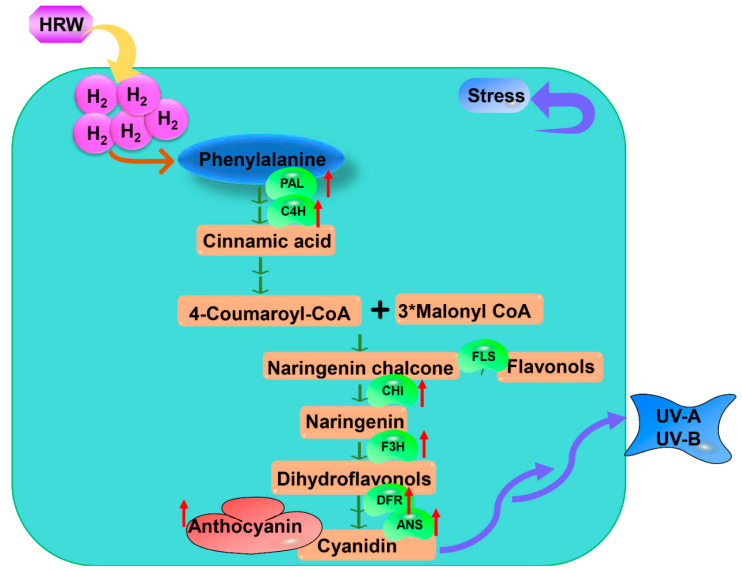
The mode of H_2_-mediated flavonoid pathway under abiotic stress. H_2_-mediated phenylpropanoid pathway promotes anthocyanin biosynthesis under abiotic stress. Some enzymes up-regulated by H_2_ are involved in this process, including PAL, C4H, FLS, CHI, F3H, DFR, and ANS. HRW, hydrogen-rich water; H_2_, Hydrogen gas; PAL, phenylalanine ammonia-lyase; C4H, cinnamic acid 4-hydroxylase; FLS, Flavonol synthase; CHI, chalcone synthase; F3H, flavanone-3-hydroxylase; DFR, Dihydroflavonol 4 reductase; ANS, anthocyanidin synthase; UV-A, Ultraviolet-A; UV-B, Ultraviolet-B.

Flavonoids are recognized as compounds with potential health benefits due their valuable nutritional antioxidant activities and are typical substances that plant produce in response to environmental stresses. Anthocyanin is a flavonoid, and its biosynthesis pathway is well described in plants. It is derived from phenylalanine, catalyzed by phenylalanine ammonialyase (PAL). Then it is mediated by a common step with chalcone synthase (CHS), chalcone isomerase (CHI), and flavanone 3-hydroxylase (F3H) and fluxed into anthocyanin biosynthesis by dihydroflavonol 4-reductase (DFR) and anthocyanidin synthase (ANS). Su et al. [30] showed that H_2_ could up-regulate UV-A-induced anthocyanin biosynthesis-related genes by molecular analyses, as cyanidin content was enhanced significantly. They also implied that H_2_ could alleviate UV-A irradiation by the anthocyanin accumulation pathway [30] (Table 1, Figure 3). Xie et al. [42] identified 40 (iso)flavonoids under UV-B irradiation by using ultra performance liquid chromatography-mass spectrometric (UPLC-MS), and H_2_ remarkably increased 22 of them including afromosin, afromosin 7-*O*-ß-D-glucoside-malonate, daidzein, formononetin 7-O-*ß*-D-glucoside-6″-O-malonate, garbanzol, matteucin, naringenin and so on. H_2_ significantly up-regulated UV-B-induced upregulation in the expression levels of (iso)flavonoids biosynthetic-related genes, suggesting that H_2_ conferred tolerance to UVB irradiation by regulating the (iso)flavonoids metabolism pathway [42] (Table 1, Figure 3). Information is lacking on whether flavonoids are involved in the H_2_-mediation of other stress in plants.

### 3.8. HO-1 Signaling

**Figure 4:**
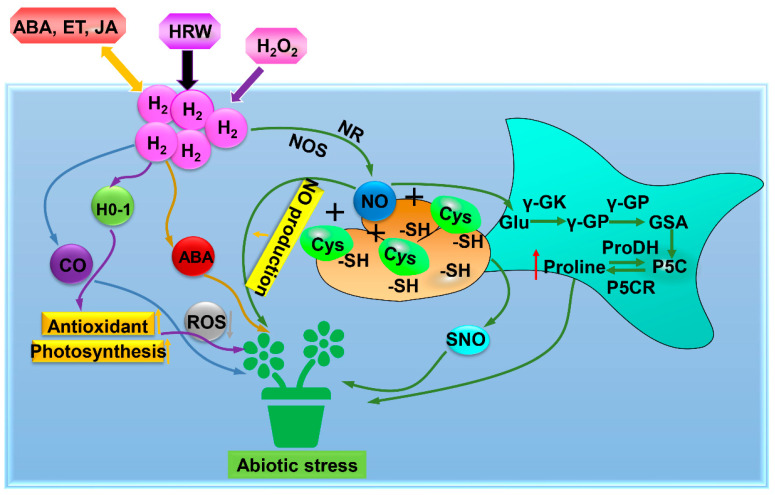
The mode of H_2_-mediated HO-1 signaling, NO, CO, and plant hormone action under abiotic stress. Under abiotic stress, H_2_ could relieve abiotic stresses by *S*-nitrosylation, inducing proline synthesis and re-establishing redox balance. H_2_ could also regulate the antioxidant system to relieve abiotic stresses in plants. Besides, H_2_ responded to abiotic stresses by modulating ABA, ethylene, or jasmonate acid hormone signaling pathways. ABA, abscisic acid; ET, Ethylene; JA, Jasmonic acid; HRW, hydrogen-rich water; H_2_O_2_, Hydrogen peroxide; H_2_, Hydrogen gas; CO, carbon monoxide; HO-1, Heme oxygenase-1; ROS, Reactive oxygen species; NOS, nitric oxide synthase; NR, nitrate reductase; NO, nitric oxide; Cys, Cysteine; -SH, sulfhydryl group; SNO, *S*-nitrosylation; Glu, Glutamate; γ-GK, Glutamyl kinase; γ-GP, Glutamyl phosphate; GSA, Glutamyl semialdehyde; P5C, Pyrrolin-5-carboxylic acid; P5CR, Pyrroline 5-carboxylate reductase; Proline, Proline dehydrogenase.

H_2_ or the heme oxygenase-1 (HO-1) inducer hemin could reduce lipid peroxidation, O_2_^−^ and H_2_O_2_ levels which enhanced tolerance of alfalfa seedlings to oxidative stress induced by PQ [6] (Table 1, Figure 4). Further results indicated that H_2_ dramatically regulated the gene expression of *MsHO-1*, corresponding protein expression and HO-1 activity, suggesting that H_2_ improved the PQ-induced oxidative stress tolerance of alfalfa seedlings by regulating HO-1 signaling [6]. Under PEG-induced osmotic stress, exogenously applied H_2_O_2_ could mimic the protective effect of H_2_ on alfalfa seedlings [11] (Table 1, Figure 4). H_2_- and H_2_O_2_-induced activities of SOD, POD and APX enzymes could be inhibited by HO-1 inhibitor ZnPP, suggesting that H_2_O_2_ might be involved in H_2_-mediated osmotic stress via HO-1 signaling [47] (Table 1, Figure 4). However, the exact mechanisms by which this process occurs are not yet known.

### 3.9. NO, CO, and Plant Hormones

Chen et al. [38] reported that Al-induced inhibition of alfalfa root growth was alleviated when NO was removed by a NO scavenger followed by decreasing NO production, suggesting that H_2_ alleviated Al stress by NO signaling (Table 1, Figure 4). Proline synthesis and antioxidant defense were stimulated by H_2_ and NO under osmotic stress [46] (Table 1, Figure 4). H_2_-triggered *S*-nitrosylation was inhibited when endogenous NO was removed [46]. Therefore, they suggested the involvement of NO in H_2_-triggered osmotic tolerance by *S*-nitrosylation, inducing proline synthesis and re-establishing redox balance [46]. Chen et al. [39] indicated the involvement of CO in H_2_-induced adventitious rooting under drought stress via activating antioxidant enzymes, reducing TBARS, O_2_^−^-, and H_2_O_2_ levels, enhancing RWC and photosynthetic capacity (Table 1, Figure 4). H_2_ may have an effect on Cd and salt stress in rice by modulating ABA, ethylene, or jasmonate acid hormone signaling pathways [12] (Table 1, Figure 4). Jin et al. [47] revealed that H_2_ as a positive regulator alleviated drought stress through ABA and modulation of apoplastic pH. However, the interaction roles of H_2_ and other signalling molecules under abiotic stress remain unclear (Table 1, Figure 4). Further research is required to investigate the interaction between H_2_ and other signaling molecules in alleviating abiotic stress.

## 4. Modulation of Gene Expression by H_2_ under Abiotic Stress

Some gene activity must inevitably be changed when H_2_ alleviates environmental stresses. At the transcript level, H_2_-upregulated the relative expression of antioxidant defense system-related genes *SOD*, *CAT*, *GR* and *noxR* to improve tolerance of CdCl_2_ and NaCl in *Hypsizygus marmoreus* [44] (Table 2). H_2_ promoted anthocyanin accumulation via exerting different effects on the expression levels of flavonoid-related genes *PAL*, *CHS*, *CHI*, *F3H*, *DFR* and *ANS* under UV-A [30] (Table 2). Under PQ-induced oxidative stress, H_2_ could dramatically increase levels of the antioxidant defense system-related genes *Cu/Zn-SOD*, *Mn-SOD*, *POD*, *APX2* and *HO-1* transcript and HO-1 protein [6] (Table 2). Wu et al. [20] reported that improvement of Cd tolerance by H_2_ was closely associated with H_2_-upregulated expression levels of the antioxidant defense system-related genes *IRT1*, *IRT2*, *Nramp1*, *HAM2*, *HAM3* and *HAM4* in Chinese cabbage (Table 2). H_2_ up-regulated significantly the expression levels of ROS and antioxidant defense system and ion homeostasis-related genes *Cu*, *Zn-SOD*, *Mn-SOD*, *POD*, *APX1/2*, *GPX*, *ECS*, *GS*, *hGS* and *GR1/2* under Cd stress [45] (Table 2). Direct exposure to H_2_ via treatment with HRW induced the expression of ROS and antioxidant defense system-related genes *POD*, *APX1/2*, *MDHAR*, *ECS*, *GS* and *GR1*, suggesting that H_2_ response to Hg stress entailed regulating the expression of ROS and antioxidant defense system-related genes [43] (Table 2). *miR398* transcripts were downregulated by H_2_, and the expression levels of its targets *Cu/Zn-SOD1* (*CSD1*) and *Cu/Zn-SOD2* (*CSD2*) were increased [29] (Table 2). By contrast, miR319 transcripts were differentially increased, showing a relatively negative correlation with its target genes *PROLIFERATING CELL FACTOR5* (*PCF5*) and *PROLIFERATING CELL FACTOR8* (*PCF8*) [29]. Recently, Dai et al. [19] indicated that H_2_ alleviated the detrimental effects of Cd stress on the growth of alfalfa seedlings by upregulating the expression levels of antioxidant defense system and glutathione homeostasis-related genes *IDH*, *Cu*, *Zn-SOD*, *NAD-dependent aldehyde dehydrogenase*, *Amine oxidase*, Cysteine desulfurase, *Peroxidase* and *Cysteine synthase* and downregulating the expression levels of ion homeostasis and nutrient element homeostasis-related genes *Phosphoenolpyruvate carboxylase* and *Ferritin* (Table 2). H_2_ enhanced heat resistance by upregulating the expression of heat response gene *HSP70* in cucumber leaves [28] (Table 2). In Arabidopsis, genetic evidence suggested that *SOS1* and *cAPX1* might be the target genes of H_2_ signaling response to salt stress [5] (Table 2). H_2_ also modulated genes/proteins of zinc-finger transcription factor ZAT10/12 in Arabidopsis under salt stress [5]. As described above, H_2_ improved the stress tolerance of plants by regulating the transcription of some specific genes, including ROS and the antioxidant defense system, including flavonoid, ion homeostasis, glutathione homeostasis, and nutrient element homeostasis-related genes. Although these specific genes may function in H_2_ metabolism, their regulation network remains unclear.

## 5. Conclusions and Perspectives

Abiotic stresses have caused great threats to plant growth and development. The effects of H_2_ on abiotic stresses have drawn more and more attention. Accumulating evidence presented in this review indicates that H_2_ is one of the important signaling molecules alleviating various abiotic stresses, including temperature, osmotic stress, light, PQ-induced oxidative stress, and metal stresses. The regulation of the antioxidant defense system, flavonoid pathway, photosynthetic capacity, re-established ion homeostasis, re-established glutathione homeostasis, maintained nutrient element homeostasis, glucose metabolism, and HO-1, NO, CO and plant hormone signaling by H_2_ are essential strategies to alleviate abiotic stresses. The expression levels of some related genes are also regulated by H_2_ under abiotic stresses.

Although it is now well-known that H_2_ confers tolerance to various abiotic stresses in plants, many pieces of the puzzle are still missing. The regulatory mechanisms associated with its responses to environmental stimuli are still a subject of great interest. Further research is required to explore the physiological and molecular mechanisms underlying the role of H_2_ in modulating plant abiotic and biotic stresses. Very little is known about the relationship between H_2_ and other signaling molecules or plant hormones in response to abiotic stresses. Future studies regarding stress resistance enhanced by H_2_ should focus on the interaction between H_2_ with other signaling molecules or plant hormones. Simultaneously, it will be important to explore whether H_2_ increases resistance to pests and diseases or improves the yield and quality of crop plants. Although H_2_ is now the subject of increasing research effort, there are many challenges to address.

## Figures and Tables

**Table 2:** Overview of H_2_-mediated genes under abiotic stresses in plants.

Plants	Tissues	Genes	Stresses	References
*Hypsizygus marmoreus*	/	*SOD*, *CAT*, *GR*, *noxR*	Cd/Salt	[44]
*Radish*	Hypocotyls	*PAL*, *CHS*, *CHI, F3H, DFR*, *ANS*	UV-A	[30]
*Alfalfa*	Leaves	*Cu/Zn-SOD*, *Mn-SOD*, *POD*, *APX2*, *HO-1*	PQ	[6]
Chinese cabbage	Roots	*IRT1*, *IRT2*, *Nramp1*, *HAM2*, *HAM3*, *HAM4*	Cd	[20]
*Alfalfa*		*Cu*, *Zn-SOD*, *Mn-SOD*, *POD*, *APX1/2*, *GPX*, *ECS*, *GS*, *hGS*, *GR1/2*	Cd	[45]
		*POD*, *APX1/2*, *MDHAR*, *ECS*, *GS*, *GR1*	*Hg*	[43]
*Rice*	Seedling	*CSD1*, *CSD2, PCF, PCF8*	Cold	[29]
*Alfalfar*		*IDH, Cu, Zn-SOD, NAD-dependent aldehyde dehydrogenase, Amine oxidase, Cysteine desulfurase, Peroxidase, Cysteine synthase, Phosphoenolpyruvate carboxylase, Ferritin*	Cd	[19]
Arabidopsis	Roots/Leaves	*SOS1, cAPX1, ZAT10/12*	Salt	[5]

Notes: PQ, paraquat; UV-A, Ultraviolet-A; Hg, Mercury; Cd, Cadmium.

## Data Availability

Not applicable.

## References

[1] Huang C.S., Kawamura T., Toyoda Y., Nakao A. (2010). Recent advances in hydrogen research as a therapeutic medical gas. Free Radic. Res..

[2] Stephenson M., Stickland L.H. (1931). Hydrogenase: A bacterial enzyme activating molecular hydrogen: The properties of the enzyme. Biochem. J..

[3] Gaffron H., Rubin J. (1942). Fermentative and photochemical production of hydrogen in algae. J. Gen. Physiol..

[4] Sanadze G.A. (1961). Absorption of molecular hydrogen by green leaves in light. Fiziol Rast.

[5] Xie Y.J., Mao Y., Lai D.W., Zhang W., Shen W.B. (2012). H_2_ enhances Arabidopsis salt tolerance by manipulating ZAT10/12-mediated antioxidant defence and controlling sodium exclusion. PLoS ONE.

[6] Jin Q.J., Zhu K.K., Cui W.T., Xie Y.J., Han B., Shen W.B. (2013). Hydrogen gas acts as a novel bioactive molecule in enhancing plant tolerance to paraquat-induced oxidative stress via the modulation of heme oxygenase-1 signalling system. Plant Cell Environ..

[7] Tamagnini P., Axelsson R., Lindberg P., Oxelfelt F., Wünschiers R., Lindblad P. (2002). Hydrogenases and hydrogen metabolism of cyanobacteria. Microbiol. Molecul. Biol. R..

[8] Das D., Khanna N., Veziroglu T.N. (2008). Recent developments in biological hydrogen production processes. Chem. Ind. Chem. Eng. Q..

[9] Kim D.H., Kim M.S. (2011). Hydrogenases for biological hydrogen production. Bioresour. Technol..

[10] Wu Q., Huang L.P., Su N.N., Shabala L., Wang H.Y., Huang X., Wen R.Y., Yu M., Cui J., Shabala S. (2020). Calcium-dependent hydrogen peroxide mediates hydrogen-rich water-reduced cadmium uptake in plant roots. Plant Physiol..

[11] Jin Q.J., Cui W.T., Dai C., Zhu K.K., Zhang J., Wang R., Shen W.B. (2016). Involvement of hydrogen peroxide and heme oxygenase-1 in hydrogen gas-induced osomotic stress tolerance in alfalfa. Plant Growth Regul..

[12] Zeng J.Q., Zhang M.Y., Sun X.J. (2013). Molecular hydrogen is involved in phytohormone signaling and stress responses in plants. PLoS ONE.

[13] Ming Y., Ma Q.H., Han X.L., Li H.Y. (2020). Molecular hydrogen improves type 2 diabetes through inhibiting oxidative stress. Exp. Ther. Med..

[14] Yuan L.J., Shen J.L. (2016). Hydrogen, a potential safeguard for graft-versus-host disease and graft ischemia-reperfusion injury?. Clinics.

[15] Li S., Liao R.R., Sheng X.Y., Luo X.J., Zhang X., Wen X.M., Zhou J., Peng K. (2019). Hydrogen gas in cancer treatment. Front. Oncol..

[16] Vacek T.P., Rehman S., Neamtu D., Yu S., Givimani S., Tyagi S.C. (2015). Matrix metalloproteinases in atherosclerosis: Role of nitric oxide, hydrogen sulfide, homocysteine, and polymorphisms. Vasc. Health Risk Manag..

[17] Qian L., Shen J., Sun X., Sun X., Ohta S., Nakao A. (2015). Therapeutic effects of hydrogen on different diseases. Hydrogen Molecular Biology and Medicine.

[18] Xu S., Zhu S.S., Jiang Y.L., Wang N., Wang R., Shen W.B., Yang J. (2013). Hydrogen-rich water alleviates salt stress in rice during seed germination. Plant Soil.

[19] Dai C., Cui W.T., Pan J.C., Xie Y.J., Wang J., Shen W.B. (2017). Proteomic analysis provides insights into the molecular bases of hydrogen gas-induced cadmium resistance in *Medicago sativa*. J. Proteom..

[20] Wu Q., Su N.N., Cai J.T., Shen Z.G., Cui J. (2015). Hydrogen-rich water enhances Cadmium in Chinese cabbage by reducing cadmium uptake and increasing antioxidant capacities. J. Plant Physiol..

[21] Zhang X.N., Zhao X.Q., Wang Z.Q., Shen W.B., Xu X.M. (2015). Protective effects of hydrogen-rich water on the photosynthetic apparatus of maize seedlings (*Zea mays* L.) as a result of an increase in antioxidant enzyme activities under highlight stress. Plant Growth Regul..

[22] Lin Y.T., Zhang W., Qi F., Cui W.T., Xie Y.J., Shen W.B. (2014). Hydrogen-rich water regulates cucumber adventitious root development in a heme oxygenase-1/carbon monoxide-dependent manner. J. Plant Physiol..

[23] Zhu Y.C., Liao W.B., Wang M., Niu L.J., Xu Q.Q., Jin X. (2016). Nitric oxide is required for hydrogen gas-induced adventitious root formation in cucumber. J. Plant Physiol..

[24] Hu H.L., Li P.X., Wang Y.N., Gu R.X. (2014). Hydrogen-rich water delays postharvest ripening and senescence of kiwifruit. Food Chem..

[25] Apel K., Hirt H. (2004). Reactive oxygen species: Metabolism, oxidative stress and signal transduction. Annu. Rev. Plant Biol..

[26] Farooq M., Wahid A., Kobayashi N., Fujita D., Basra S.M.A. (2009). Plant drought stress: Effects, mechanisms and management. Agron. Sustain. Dev..

[27] Zhao X.Q., Chen Q.H., Wang Y.M., Shen Z.G., Shen W.B., Xu X.M. (2017). Hydrogen-rich water induces aluminum tolerance in maize seedlings by enhancing antioxidant capacities and nutrient homeostasis. Ecotoxicol. Environ. Saf..

[28] Chen Q.H., Zhao X.Q., Lei D.K., Hu S.B., Shen Z.G., Shen W.B., Xu X.M. (2017). Hydrogen-rich water pretreatment alters photosynthetic gas exchange, chlorophyll fluorescence, and antioxidant activities in heat-stressed cucumber leaves. Plant Growth Regul..

[29] Xu S., Jiang Y.L., Cui W.T., Jin Q.J., Zhang Y.H., Bu D., Fu J.Y., Wang R., Zhou F., Shen W.B. (2017). Hydrogen enhances adaptation of rice seedlings to cold stress via the reestablishment of redox homeostasis mediated by miRNA expression. Plant Soil.

[30] Su N.N., Wu Q., Liu Y.Y., Cai J.T., Shen W.B., Xia K., Cui J. (2014). Hydrogen-rich water re-establishes ROS homeostasis but exerts differential effects on anthocyanin synthesis in two varieties of radish sprouts under UV-A irradiation. J. Agr. Food Chem..

[31] Nakashima K., Yamaguchi-Shinozaki K. (2013). ABA signaling in stress-response and seed development. Plant Cell Rep..

[32] Li B.Q., Zhang C.F., Cao B.H., Qin G.Z., Wang W.H., Tian S.P. (2012). Brassinolide enhances cold stress tolerance of fruit by regulating plasma membrane proteins and lipids. Amino Acids..

[33] Cao Y.R., Chen S.Y., Zhang J.S. (2008). Ethylene signaling regulates salt stress response: An overview. Plant Signal. Behav..

[34] Siddiqui M.H., Al-Whaibi M.H., Basalah M.O. (2011). Role of nitric oxide in tolerance of plants to abiotic stress. Protoplasma.

[35] Hancock J.T. (2019). Hydrogen sulfide and environmental stresses. Environ. Exp. Bot..

[36] He H.Y., He L.F. (2014). The role of carbon monoxide signaling in the responses of plants to abiotic stresses. Nitric Oxide.

[37] Piantadosi C.A. (2008). Carbon monoxide, reactive oxygen signaling, and oxidative stress. Free Radic. Biol. Med..

[38] Chen M., Cui W.T., Zhu K.K., Xie Y.J., Zhang C.H., Shen W.B. (2014). Hydrogen-rich water alleviates aluminum-induced inhibition of root elongation in alfalfa via decreasing nitric oxide production. J. Hazard. Mater..

[39] Chen Y., Wang M., Hu L.L., Liao W.B., Dawuda M.M., Li C.L. (2017). Carbon monoxide is involved in hydrogen gas-induced adventitious root development in cucumber under simulated drought stress. Front. Plant Sci..

[40] Zhu J.K. (2016). Abiotic stress signaling and responses in plants. Cell.

[41] Asada K. (2006). Production and scavenging of reactive oxygen species in chloroplasts and their functions. Plant Physiol..

[42] Xie Y.J., Wei Z., Duan X.L., Dai C., Zhang Y.H., Cui W.T., Wang R., Shen W.B. (2015). Hydrogen-rich water-alleviated ultraviolet-B-triggered oxidative damage is partially associated with the manipulation of the metabolism of(iso)flavonoids and antioxidant defence in *Medicago sativa*. Funct. Plant Biol..

[43] Cui W.T., Fang P., Zhu K.K., Mao Y., Gao C.Y., Xie Y.J., Wang J., Shen W.B. (2014). Hydrogen-rich water confers plant tolerance to mercury toxicity in alfalfa seedlings. Ecotox. Environ. Saf..

[44] Zhang J.J., Hao H.B., Chen M.J., Wang H., Feng Z.Y., Chen H. (2017). Hydrogen-rich water alleviates the toxicities of different stresses to mycelial growth in *Hypsizygus marmoreus*. AMB Expr..

[45] Cui W.T., Gao C.Y., Fang P., Lin G.Q., Shen W.B. (2013). Alleviation of cadmium toxicity in Medicago sativa by hydrogen-rich water. J. Hazard. Mater..

[46] Su J.C., Zhang Y.H., Nie Y., Cheng D., Wang R., Hu H.L., Chen J., Zhang J.F., Du Y.W., Shen W.B. (2018). Hydrogen-induced osmotic tolerance is associated with nitric oxide-mediated proline accumulation and reestablishment of redox balance in alfalfa seedlings. Environ. Exp. Bot..

[47] Jin Q.J., Zhu K.K., Cui W.T., Li L.N., Shen W.B. (2016). Hydrogen-modulated stomatal sensitivity to abscisic acid and drought tolerance via the regulation of apoplastic pH in Medicago sativa. J. Plant Growth Regul..

[48] Mittler R., Vanderauwera S., Gollery M., Van Breusegem F. (2004). Reactive oxygen gene network of plants. Trends Plant Sci..

[49] Mittler R. (2002). Oxidative stress, antioxidants and stress tolerance. Trends Plant Sci..

[50] Foyer C.H., Noctor G. (2005). Redox homeostasis and antioxidant signaling: A metabolic interface between stress perception and physiological responses. Plant Cell.

[51] Kaushik D., Aryadeep R. (2014). Reactive oxygen species (ROS) and response of antioxidants as ROS-scavengers during environmental stress in plants. Front. Environ. Sci..

[52] Demmig-Adams B., Adams W.W., Barker D.H., Logan B.A., Bowling D.R., Verhoeven A.S. (1996). Using chlorophyll fluorescence to assess the fraction of absorbed light allocated to thermal dissipation of excess excitation. Physiol. Plant.

